# 

**Published:** 1861-05

**Authors:** 


### CONTENTS.

ORIGINAL ESSAYS AND COMMUNICATIONS.

Impartation. By Dr. W. H. Atkinson.......... 261
Professional Success. By A. S. Talbert, D. D. S., Lexington, Ky.......... 276
Diagnosis. By Dr. W. H. Atkinson.......... 282
Who are Dentists. By Wm. A. Pease.......... 291

PROCEEDINGS OF SOCIETIES.

Kentucky State Dental Association.......... 297

SELECTIONS.

Death from Anæsthesia.......... 302
Epithelial Cancer of the Lip.......... 306
The Annoyances of the Administration of Chloroform.......... 307
On Chloroform and the Narcotic Remedial Series.......... 308
Stramonium in Neuralgia.......... 317
Action of Different Medicines on the Mental Faculties.......... 318
Extracts from the Proceedings of the Wayne County Medical Society.......... 318
Editorial.......... 320

